# Characterization and Toxicity of Crude Toxins Produced by *Cordyceps fumosorosea* against *Bemisia tabaci* (Gennadius) and *Aphis craccivora* (Koch)

**DOI:** 10.3390/toxins13030220

**Published:** 2021-03-18

**Authors:** Jianhui Wu, Bo Yang, Jing Xu, Andrew G. S. Cuthbertson, Shaukat Ali

**Affiliations:** 1 Key Laboratory of Bio-Pesticide Innovation and Application, Guangzhou 510642, China; jhw@scau.edu.cn (J.W.); yb@stu.scau.edu.cn (B.Y.); zhibaoxujing@stu.scau.edu.cn (J.X.); 2 Engineering Research Center of Biological Control, Ministry of Education and Guangdong Province, South China Agricultural University, Guangzhou 510642, China; 3 Independent Science Advisor, York YO10 5AQ, UK; andrew_cuthbertson@live.co.uk

**Keywords:** biological control, *Cordyceps fumosorosea*-SP502, toxin, whitefly, aphids, toxicity

## Abstract

*Cordyceps fumosorosea*, an insect pathogenic fungus, produces different toxins/secondary metabolites which can act as pest control agents. This study reports the extraction and characterization of crude mycelial extracts of *C. fumosorosea* isolate SP502 along with their bio-efficacy against *Bemisia tabaci* and *Aphis craccivora*. Fourier transform infrared spectroscopy, liquid chromatography, mass spectrometery and nuclear magnetic resonance analysis of *C. fumosorosea* isolate SP502 extracts showed the presence of five major compounds—Trichodermin, 5-Methylmellein, Brevianamide F, Enniatin and Beauvericin—which all may potentially be involved in insecticidal activity. The HPLC analysis of *C. fumosorosea* mycelial extracts and Beauvericin standard showed similar chromatographic peaks, with the content of Beauvericin in the crude toxin being calculated as 0.66 mg/ml. The median lethal concentrations of *C. fumosorosea* mycelial extracts towards first, second, third and fourth instar nymphs of *A. craccivora* were 46.35, 54.55, 68.94, and 81.92 µg/mL, respectively. The median lethal concentrations of *C. fumosorosea* mycelial extracts towards first, second, third and fourth instar nymphs of *B. tabaci* were 62.67, 72.84, 77.40, and 94.40 µg/mL, respectively. Our results demonstrate that bioactive compounds produced by *C. fumosorosea* isolate SP502 have insecticidal properties and could, therefore, be developed into biopesticides for the management of *B. tabaci* and *A. craccivora*.

## 1. Introduction

*Bemisia tabaci* Gennadius (Hemiptera: Aleyrodidae), a species complex containing 36 or more cryptic species, continues to be a major threat to field crop production across the globe [1,2]. Population levels and infestation by *B. tabaci* Middle East-Asia Minor 1 (MEAM1) (previously known as “Biotype B”) is by far the most common. It is the most notoriously invasive species of the *Bemisia* species complex, having increased greatly during the last twenty years [3,4]. *Bemisia tabaci* MEAM1 damages plants directly through feeding on cell sap, and indirectly by honey dew secretion which leads to sooty mould growth on plant surfaces [5]. In addition, *B. tabaci* MEAM1 is also a vector of over 150 viral crop diseases [6].

*Aphis craccivora* Koch (Homoptera: Aphididae), also known as cowpea aphid, is a major pest of cowpea and other crops in different regions of the world [7]. Both nymphs and adults can damage the crops. *Aphis craccivora* causes economic damage either by directly sucking the sap from aerial plant parts or by releasing honeydew on plant leaves which leads to sooty mould production [8]. The severe damage of *A. craccivora* can cause delayed flowering and yield a loss of more than 50% [9]. In addition, they are also a vector of many viral plant diseases [10].

Insect pest management has generally been achieved using synthetic agro-chemicals. However, their overuse has resulted in the development of insecticide resistance by many insect pests [11,12]. In addition, the negative impacts of synthetic insecticides on beneficial insects [13,14] and humans have forced the agricultural industry to seek the development of bio-rational and natural/indigenous methods to control insect pests [15]. Therefore, the development and application of biochemicals/biopesticides originating from naturally occurring insect pathogenic fungi can potentially provide an alternative to synthetic insecticides [16].

Entomopathogenic fungi have been suggested as potential agents for the biological control of different insects for over a century. Diversified species range, complex metabolic types, and appropriate safety levels for humans and other non-target organisms of entomopathogenic fungi makes them more attractive than other pest control tactics [15]. *Cordyceps fumosorosea* (previously known as *Isaria fumosorosea*) is a well-documented entomopathogen used for insect pest management [17]. Different isolates of *C. fumosorosea* have been effective against aphids, scales, whiteflies and other insect pests across the globe [17,18,19,20,21]. *Cordyceps fumosorosea* produces different secondary compounds/toxins such as Beauvericin, Beauverolides and 2,6-pyridindicarboxylic acid (also known as dipicolinic acid; DPA) [22,23,24,25] which may be effective in the control of insect pests.

The aims of the current study were to (i) extract and characterise the mycelial extracts/secondary chemicals of *C. fumosorosea* isolate SP502 which has shown considerable pathogenicity against *B. tabaci* in our previous studies [26]; (ii) undertake toxicity assays of mycelial extracts/secondary chemicals against nymphal instars of *B. tabaci* and *A. craccivora.*

## 2. Results

### 2.1. Fourier Transformed Infrared Spectroscopy (FTIR)

**Figure 1:**
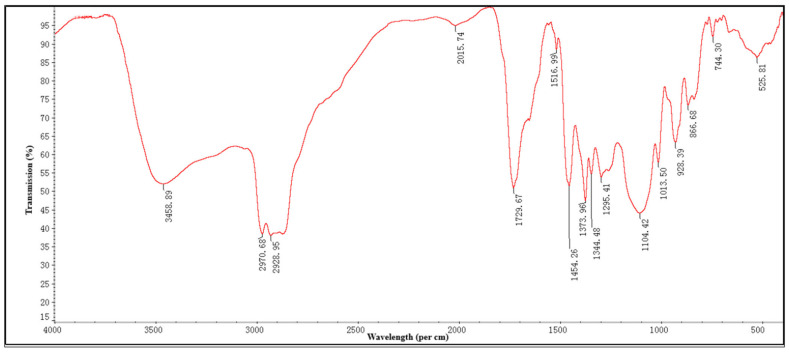
FTIR analysis of the ethyl acetate mycelia extract obtained from *Cordyceps fumosorosea*.

FTIR analysis was performed to find the functional groups of the active compounds based on the peak value in the infrared region (Figure 1 and Table 1). FTIR analysis showed the presence of prominent bands due to the O-H stretch alcohols or phenols (3458.89 cm^−1^), N-H alkane (Methyl) (2970.68 cm^−1^), C-H alkane (Methylene) (2928.95 cm^−1^), C≡C stretch nitrile (2015.74 cm^−1^), C=O carboxylic acid (1729.67 cm^−1^), C=C stretch alkane (1516.99 cm^−1^), C=O trans-alkenes (1454.26 cm^−1^), C=C trans-alkenes (1373.96 cm^−1^), C=C trans-alkenes (1344.48 cm^−1^), C=C ester carbonyl group (1295.41 cm^−1^), C=C ether/alcohol (1104.42 cm^−1^), C-O trans-alkenes (1013.50 cm^−1^), C=C vinyl-alkenes (928.39 cm^−1^), C=C aromatics (866.68; 744.30 cm^−1^), and aromatics (Ring) (525.81 cm^−1^).

### 2.2. Liquid Chromatography-Mass Spectrometry Analysis of Cordyceps fumosorosea 

**Figure 2:**
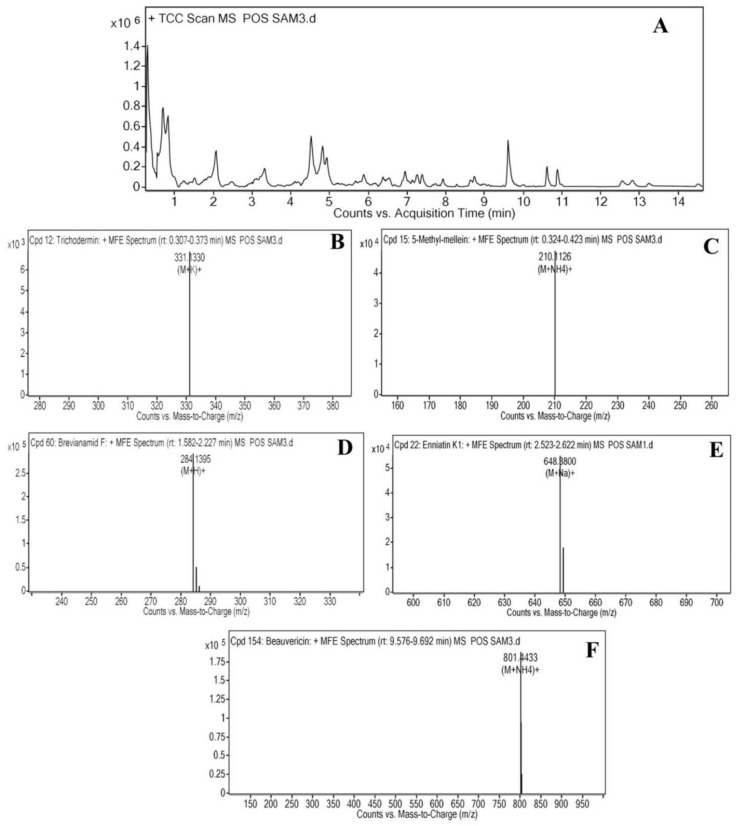
LC-Ms analysis of bioactive compounds produced by *Cordyceps fumosorosea* isolate SP502. (**A**) LC-MS profile; (**B**) Trichodermin; (**C**) 5-Methylmellein; (**D**) Brevianamide F; (**E**) Enniatin; (**F**) Beauvericin.

The LC-MS results obtained from the ethyl acetate extract of SP502 indicated the presence of five main fractions: Trichodermin (C_17_H_24_O_4_; retention time: 0.340), 5-Methylmellein (C_11_H_13_O_3_; retention time: 0.344), Brevianamide F (C_16_H_17_N_3_O_2_; retention time: 2.0621), Enniatin (C_32_H_55_N_3_ O_9_; retention time: 2.565) and Beauvericin (C_45_H_57_N_3_ O_9_; retention time: 9.615) which may potentially be involved in insecticidal activity (Figure 2 and Table 2).

### 2.3. Nuclear Magnetic Resonance (NMR)

**Figure 3:**
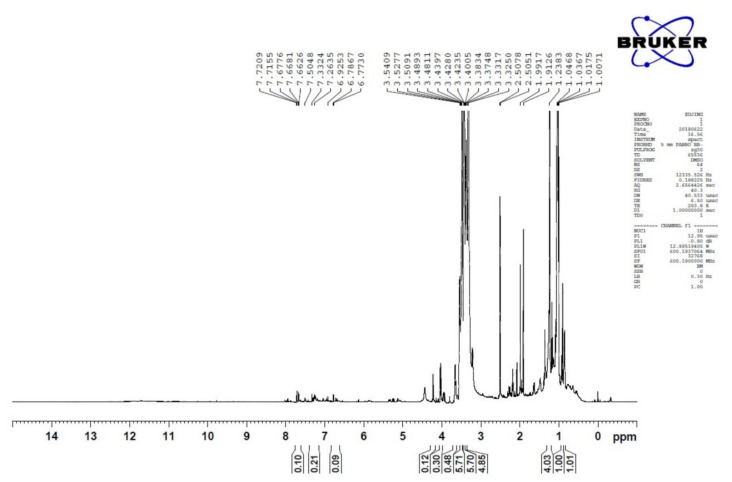
The ^1^H NMR analysis of bio-active compounds produced by *Cordyceps fumosorosea* isolate SP502.

The ^1^H NMR analysis of metabolic compounds produced by *C. fumosorosea* isolate SP502 shows a high resonance band in aliphatic (0.9–1.4 ppm), allylic, pyrrolidine ring and NeCH (2.0–2.6 ppm and 2.1–3.5 ppm), an ether linkage (3.5–4.3 ppm), and amide proton (7.3–7.8 ppm) regions. These findings further confirm the production of metabolic compounds detected through LC-MS analysis (Figure 3).

### 2.4. HPLC Analysis

**Figure 4:**
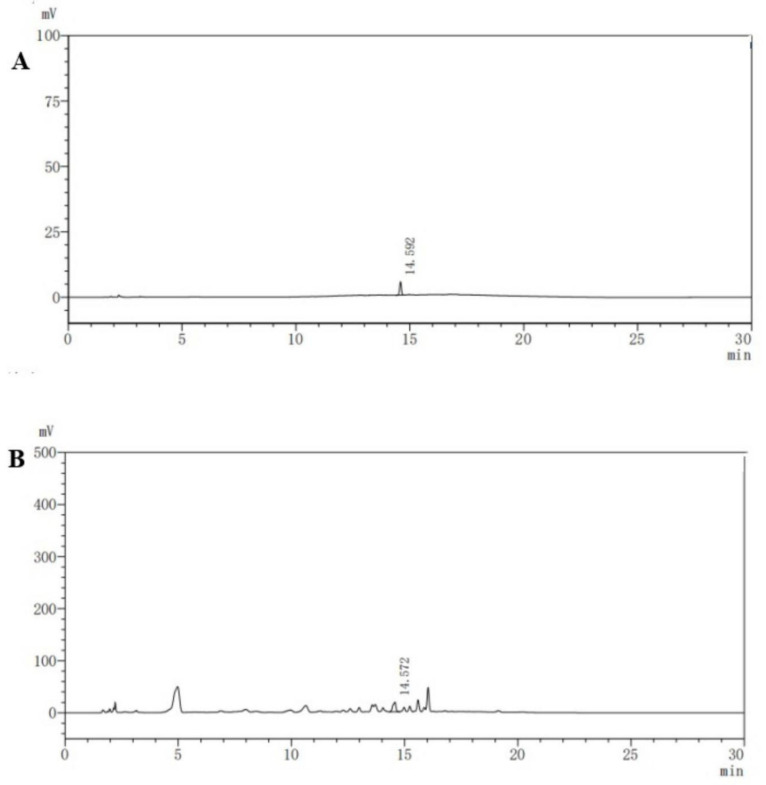
(**A**) The HPLC chromatogram of Beauvericin standard and (**B**) the HPLC chromatogram of mycelial extracts obtained from *Cordyceps fumosorosea.*

The HPLC analysis of *C. fumosorosea* mycelial extracts and Beauvericin standard showed a similar chromatographic peak (at retention time 14.572 and 14.592 min). The content of Beauvericin in crude toxin was calculated to be 0.66 mg/mL (Figure 4).

### 2.5. Toxicity of C. fumosorosea Secondary Metabolites Against Immature Instars of B. tabaci and A. craccivora

The insecticidal activity of ethyl acetate mycelial extracts from *C. fumosorosea* was tested against different nymphal instar stages of *B. tabaci* and *A. craccivora* under laboratory conditions. The mycelial extracts were toxic against first to fourth instar nymphs of *A. craccivora* after 3 days of exposure with LC_50_ values of 46.35, 54.55, 68.94, and 81.92 µg/mL against first, second, third and fourth instar nymphs, respectively (Table 3).

The bioactive compounds produced by *C. fumosorosea* were also toxic against first to fourth instar nymphs of *B. tabaci* after 3 days of exposure with LC_50_ values of 62.67, 72.84, 77.40, and 94.40 µg/mL against first, second, third and fourth instar nymphs, respectively (Table 4).

## 3. Discussion

*Cordyceps fumosorosea* (previously *Isaria fumosorosea*) is a well-documented entomopathogenic fungus that has been widely commercialised for whitefly control [17]. This fungal species is also known to produce different insecticidal mycotoxins [25,27]. This study reports the characterization of mycelial extracts produced by *C. fumosorosea-*SP502 and their toxicity against nymphal instars of *B. tabaci* and *A. craccivora.*

FTIR analysis of the ethyl acetate mycelium extract showed the presence of prominent bands due to the O-H stretch alcohols or phenols, N-H alkane (Methyl), C-H alkane (Methylene), C≡C stretch nitrile, C=O carboxylic acid, C=C stretch alkane, C=O trans-alkenes, C=C trans- alkenes, C=C trans-alkenes, C=C ester carbonyl group, C=C ether/alcohol, C-O trans-alkenes, C=C vinyl-alkenes, C=C aromatics, and aromatics (Ring). Similar functional types have also been observed by Ragavendran et al. [28] and Vivekanandhan et al. [29] in the FTIR profiles of the entomopathogenic fungus, *Beauveria bassiana*. The LC-MS analysis results revealed the presence of five major compounds responsible for the toxic activity of mycelial extracts, namely Trichodermin, 5-Methylmellein, Brevianamide F, Enniatin and Beauvericin. Previously, Qasim et al. [25] reported the production of 17 different mycotoxins, including Beauvericin by *C. fumosorosea* isolate FAFU-1. Supothina et al. [30] also showed Beauvericin production by *Cordyceps tenuipes*. The ^1^H NMR analysis of metabolic compounds produced by *C. fumosorosea* isolate SP502 shows a high resonance band at the region for aliphatic, allylic, pyrrolidine ring and NeCH, an ether linkage, and amide proton. These results are similar to the ^1^H NMR spectra of *C. tenuipes* [30] and *Metarhizium anisopliae* [31]. The HPLC analysis of mycelial extracts was compared with the Beauvercin standard and they showed similar chromatographic peaks. These results are in agreement with Supothina et al. [30], who also observed Beauvericin in mycelial extracts of *Cordyceps tenuipes*.

In the present study, mycelial extracts of *C. fumosorosea-*SP502 were found to be toxic against different nymphal stages of both *B. tabaci* and *A. craccivora.* The results clearly suggested that mycelial extracts of *C. fumosorosea-*SP502 had a broad spectrum of nymphal mortality against *B. tabaci* and *A. craccivora.* These metabolites cause infection by destroying the insect cuticle layer, which can lead to their death [28]. Similar studies have also been conducted by Wang et al. [32] using mycelial extracts of another Hypocereales fungus, *Akanthomyces muscarium* (previously *Lecanicillium muscarium*), as an insecticidal agent against *B. tabaci*. They reported LC_50_ values of 111 and 216 mg/L for mycelial extracts of *Akanthomyces muscarium* V3450 and *Akanthomyces muscarium* Vp28, respectively against third instar nymphs of *B. tabaci*.

## 4. Conclusions

Our findings reveal promising results regarding the insecticidal potential of toxic compounds produced by *C. fumosorosea* isolate SP502 against *B. tabaci* and *A. craccivora*. The mycelial extracts of *C. fumosorosea*-SP502 had a broad spectrum of nymphal mortality against both pest species. The characterisation of extracts demonstrated that Beauvericin was one of the main constituents of the mycelial extracts, since it hadinsecticidal properties. These metabolites have potential to be further developed as biopesticides formulations to control whitefly and aphid pests.

## 5. Materials and Methods

### 5.1. Insect Cultures

*Bemisia tabaci* was reared on *Solanum melongena* as outlined by Wang et al. [15]. Briefly, a stock culture of whiteflies was obtained from the Engineering Research Centre of Biological Control, Ministry of Education, South China Agricultural University (SCAU), Guangzhou, China. Whiteflies were maintained on eggplants (cultured in 18.0 cm pots and kept in cages to avoid any premature infestation from whiteflies or other insects until use) placed in cages at 26 ± 1 °C, 70 ± 10% R.H., and a photoperiod of 12 h of light: 12 h of darkness.

Stock cultures of *A. craccivora* were collected from the experimental research fields of SCAU. Following collection, the insects were reared on *Vigna unguiculata* seedlings (6 weeks old) for five generations prior to the experiments, following the method of Ekesi et al. [33] at 26 ± 1 °C, 70 ± 10% R.H., and a photoperiod of 12 h of light: 12 h of darkness. 

### 5.2. Fungal Inoculum and Culture Conditions

*Cordyceps fumosorosea* strain SP502 isolated from soil was used during this study. The fungal strain was cultured on Potato Dextrose Agar (PDA) plates following the method of Ali et al. [34]. The basal fungal suspension used during the current experiments was prepared by harvesting from PDA plates (10 days old culture) with ddH_2_O containing 0.03% Tween-80 and sieving them using filter paper (Whatman no. 2; Science Kit and Boreal Laboratories, New York, NY, USA) into sterile vials. Conidia were counted using a compound microscope and a hemocytometer (0.0625 m^2^; Fuchs-Rosenthal Merck Euro Lab, Darmstadt, Germany) at 40× magnification under a phase contrast microscope in order to calibrate a suspension of 1 × 10^7^ conidia/mL.

Erlenmeyer flasks (500 mL) containing 150 mL of sterilised culture medium (per litre) consisting of 30 g of glucose, 3 g of yeast extract, 0.39 g of KH_2_PO_4_, 1.42 g of Na_2_HPO_4_·12H_2_O, 0.60 g of MgSO_4_·7H_2_O, 0.70 g of NH_4_NO_3_ and 1.00 g of KCl were inoculated with 5 mL of conidial suspension (1 × 10^7^ conidia/mL) followed by incubation at 150 rpm and 27 °C for 5 days.

### 5.3. Secondary Metabolite Extraction from Cordyceps fumosorosea

Fungal cultures (following 5 days growth) were centrifuged at 10,000 rpm, 4 °C for 10 min by using an Eppendorf 5804R centrifuge (Eppendorf, Germany) to remove the mycelium. The supernatant was further filtered through nylon filters (pore size 0.45 µm). The bio-active compounds were extracted using 1:1 ratio of ethyl acetate as solvent by sonication at room temperature for 10 min followed by incubation at 4 ℃ for 24 h. The organic phase (pale yellow colour) was separated from the fermentation liquid and then the ethyl acetate was separated from the extracts by rotary vacuum evaporation at 45 °C. The dried extracts appearing as a brownish paste were used for further analytical studies. 

### 5.4. Liquid Chromatography-Mass Spectrophotometry (LC-MS) Analysis

The LC-MS analysis was performed by using an LC-MS/MS system, consisting of a LC Agilent 1200 using a binary pump and an automatic injector, and coupled with a 3200 QTRAP^®^ AB SCIEX equipped with a Turbo-V™ source (electrospray ionisation) interface. The chromatographic separation of the compounds was conducted at 24 ± 1 °C on a reverse-phase analytical column C_18_ (3 μm, 150 × 2 mm ID) and a guard-column C_18_ (4 × 2 mm, ID; 3 μm). Mobile phases were as follows: methanol (0.1% acetic acid and 5 mM ammonium acetate) as Phase A and water (0.1% acetic acid and 5 mM ammonium acetate) as Phase B. The following gradient was used: equilibration at 90% B for 2 min, from 80% to 20% B in 3 min, 20% B for 1 min, from 20% to 10% B in 2 min, 10% B for 6 min, from 10% to 0% B in 3 min, 100% A for 1 min, from 100% to 50% A in 3 min, return to initial conditions in 2 min, and maintain at initial conditions for 2 min. The flow rate was 0.25 mL min^−1^ in all steps. Total run time was 21 min. The injection volume was 20 µL. In regard to mycotoxin analysis, the QTRAP System was used as selected reaction monitoring (SRM). The Turbo-V™ source was used in positive mode with the following settings for source/gas parameters: curtain gas (CUR) 20, ion spray voltage (IS) 5500, source temperature (TEM) 450 °C, ion source gas 1 (GS1), and ion source gas 2 (GS2) 50. The entrance potential (EP) was the same for all analytes, i.e., 10 V. Acquisition and processing data were performed using Analyst^®^ software, version 1.5.2 (AB SCIEX, Concord, ON, Canada).

### 5.5. Fourier Transformed Infrared Spectroscopy (FTIR)

Fourier transformed infrared spectroscopy analysis was performed by using an MIR8035 FTIR spectrometer (Thermo Fisher, Darmstadt, Germany). All measurements were made at a resolution of 4 cm^-1^ over a frequency range of 400 to 4000 cm^−1^. The liquid sample was loaded directly, and the spectra were recorded at room temperature.

### 5.6. Nuclear Magnetic Resonance (NMR)

Nuclear magnetic resonance (NMR) was performed using a Bruker advance III-HD 600 NMR spectrometer (Bruker, Karlsruhe, Germany) by following the method of Wang et al. [32].

### 5.7. High Performance Liquid Chromatography (HPLC) Analysis

The crude extracts and pure Beauvericin were diluted in isopropanol and subjected to high performance liquid chromatography by using a LC-20AD HPLC system (Shimadzu Chromatographic Instruments, Kyoto, Japan) and an Ultimate XB-C18 column (4.6 × 150 mm, and 5 μm) at 35 °C. Acetonitrile was employed as the mobile phase at a flow rate of 1.0 mL/min. The injection volume was 5 µL. The compound peaks were detected at 220 nm wavelength. Beauvericin was used as the standard. The amount of specific compound that resembles the standard was expressed as mg/mL.

### 5.8. Toxicity of C. fumosorosea Secondary Metabolites against Immature Instars of B. tabaci and A. craccivora 

Different concentrations of fungal extracts (500, 200, 100, 50, 20 and 10 µg/mL) were prepared by adding the extracts to ethyl acetate. Eggplant leaves infested with individual *B. tabaci* nymphal instar stages (first, second, third or fourth instar) were dipped in each concentration for 30 s following the methods of Cuthbertson et al. [35,36], respectively. The leaves were then air dried and placed in Petri dishes (ø 9 cm) with moist filter paper for moisture maintenance. *Bemisia tabaci* nymphs dipped in Matrine (15 mg/L) acted as positive control while whitefly nymphs dipped in ddH_2_O served as negative control. Four eggplant leaves with 100 *B. tabaci* nymphs of each instar stage/leaf were used in each treatment. The whole experimental setup was maintained at 25 ± 1 °C and 80 ± 5% R.H and 16:8 h (Light/Dark photoperiod). The whole experiment was performed three times (with a fresh batch of insects each time). Insect mortality was observed on a daily basis until 3 days post treatment. 

Following the same method from Cuthbertson et al. [36], cowpea (*V. unguiculata*) leaves were dipped in different concentrations of fungal extracts (500, 200, 100, 50, 20 and 10 µg/mL) for 30 s. Cowpea leaves dipped in Matrine (15 mg/L) acted as a positive control while leaves dipped in ddH_2_O served as a negative control. The leaves were air dried and placed in Petri dishes (ø 9 cm) with a moist filter paper for moisture maintenance. Fifty newly moulted bean aphids up to 12 h old (first instar) were released on the treated and control leaf respectively in a Petri dish. The whole experimental setup was maintained at 25 ± 1 °C and 80 ± 5% R.H and 16:8 h (Light/Dark photoperiod). The whole experiment was performed three times (with a fresh batch of insects each time).

### 5.9. Data Analysis 

Mortality (%) was calculated and corrected with control mortality using Abbott’s formula [37]. LC_50_ and LC_90_ values were calculated by probit analysis. All statistical analyses were performed using SAS 8.01 [38].

## Figures and Tables

**Table 1:** FTIR spectrum of ethyl acetate extract obtained from *Cordyceps fumosorosea.*

Observed Wave Numbers (cm^−1^)	Functional Group	Bonding Pattern
3458.89	O-H stretch alcohols or phenols	Strong, broad
2970.68	N-H alkane (Methyl)	Sharp
2928.95	C-H alkane (Methylene)	Strong, medium
2015.74	C≡C stretch nitrile	Weak
1729.67	C=O carboxylic acid	Strong, sharp
1516.99	C=C stretch alkane	Weak, sharp
1454.26	C=O trans alkenes	Strong, sharp
1373.96	C=C trans alkenes	Strong, sharp
1344.48	C=C trans alkenes	Weak, sharp
1295.41	C=C ester carbonyl group	Weak, medium
1104.42	C=C ether/alcohol	Strong, medium
1013.50	C-O trans-alkenes	Strong, sharp
928.39	C=C vinyl-alkenes	Strong, sharp
866.68	C=C aromatics	Strong, medium
744.30	C=C aromatics (Ring)	Weak, sharp
525.81	C=C Aromatics (Ring)	Weak, sharp

**Table 2:** LC-MS spectrum of ethyl acetate extract obtained from *Cordyceps fumosorosea.*

Sr#	Compound Name	Rt	Molecular Formula	Biological Activity
1	Trichodermin	0.340	C_17_H_24_O_4_	Fungicide; Pesticide
2	5-Methylmellein	0.344	C_11_H_13_O_3_	Antimicrobial activity
3	Brevianamide F	2.062	C_16_H_17_N_3_ O_2_	Antioxidant activity, mycotoxin
4	Enniatin	2.565	C_32_H_55_N_3_ O_9_	Antimicrobial activity
5	Beauvericin	9.615	C_45_H_57_N_3_ O_9_	Mycotoxin

**Table 3:** Insecticidal activity of bioactive compounds produced by *Cordyceps fumosorosea* isolate SP502 against *Aphis craccivora* nymphs.

Nymphal Instar	Concentration (mg/L)	Mortality (%)	LC_50_ (LCL–UCL) (mg/L)	LC_90_ (LCL–UCL) (mg/L)	χ^2^ (df)4
1st instar	ddH_2_O	3.27	46.35 (30.89–66.65)	346.83 (203.44–865.38)	24.29
	Ethyl acetate	3.67			
	Matrine	51.16			
	10	14.29			
	20	36.67			
	50	51.67			
	100	62.97			
	200	78.99			
	500	97.84			
2nd instar	ddH_2_O	2.34	54.55 (42.11–69.94)	419.39 (280.43–747.78)	11.28
	Ethyl acetate	3.33			
	Matrine	52.34			
	10	12.91			
	20	31.06			
	50	48.21			
	100	60.37			
	200	77.04			
	500	94.67			
3rd instar	ddH_2_O	2.33	68.94 (56.14–84.77)	539.42 (377.61–873.66)	7.55
	Ethyl acetate	3.19			
	Matrine	48.67			
	10	10.97			
	20	24.67			
	50	41.87			
	100	58.09			
	200	70.33			
	500	92.33			
4th instar	ddH_2_O	2.11	81.92 (73.47–91.52)	714.74 (575.59–921.65)	3.46
	Ethyl acetate	3.56			
	Matrine	47.92			
	10	10.02			
	20	23.17			
	50	37.92			
	100	54.08			
	200	68.23			
	500	87.67			

**Table 4:** Insecticidal activity of bioactive compounds produced by *Cordyceps fumosorosea* isolate SP502 against *Bemisia tabaci* nymphs.

Nymphal Instar	Concentration (mg/L)	Mortality (%)	LC_50_ (LCL–UCL) (mg/L)	LC_90_ (LCL–UCL) (mg/L)	χ^2^ (df)4
1st instar	ddH_2_O	2.01	62.67 (39.26–100.48)	339.31 (185.15–1129.67)	9.26
	Ethyl acetate	3.16			
	Matrine	54.23			
	10	13.06			
	20	21.23			
	50	38.54			
	100	53.67			
	200	82.33			
	500	100			
2nd instar	ddH_2_O	1.06	72.84 (58.88–90.51)	442.00 (311.92–712.57)	4.06
	Ethyl acetate	2.14			
	Matrine	53.18			
	10	12.18			
	20	19.36			
	50	35.87			
	100	51.67			
	200	78.23			
	500	95.08			
3rd instar	ddH_2_O	1.37	77.40 (61.89–97.56)	535.70 (365.12–913.75)	4.52
	Ethyl acetate	2.23			
	Matrine	46.72			
	10	11.5			
	20	18.29			
	50	33.33			
	100	51.33			
	200	75.67			
	500	94.33			
4th instar	ddH_2_O	1.24	94.40 (75.48–119.81)	653.27 (439.06–1139.34)	2.31
	Ethyl acetate	2.87			
	Matrine	45.91			
	10	10.6			
	20	15.06			
	50	29.33			
	100	48.16			
	200	72.34			
	500	89.17			

## Data Availability

The raw data supporting the conclusion will be made available by the corresponding author on request.
